# Performance of Two Bioelectrical Impedance Analyses in the Diagnosis of Overweight and Obesity in Children and Adolescents: The FUPRECOL Study

**DOI:** 10.3390/nu8100575

**Published:** 2016-10-04

**Authors:** Robinson Ramírez-Vélez, Jorge Enrique Correa-Bautista, Javier Martínez-Torres, Katherine González-Ruíz, Emilio González-Jiménez, Jacqueline Schmidt-RioValle, Antonio Garcia-Hermoso

**Affiliations:** 1 Centro de Estudios para la Medición de la Actividad Física «CEMA», Escuela de Medicina y Ciencias de la Salud, Universidad del Rosario, Bogotá D.C. 111221, Colombia; jorge.correa@urosario.edu.co (J.E.C.-B.); javiermartinezt@usantotomas.edu.co (J.M.-T.); 2 Grupo de Ejercicio Físico y Deportes, Vicerrectoría de Investigaciones, Universidad Manuela Beltrán, Bogotá D.C. 110231, Colombia; Katherine.gonzalez@docentes.umb.edu.co; 3 Grupo CTS-436, Adscrito al Centro de Investigación Mente, Cerebro y Comportamiento (CIMCYC), Departamento de Enfermería, Universidad de Granada, Granada 18071, España; emigoji@ugr.es (E.G.-J.); jschmidt@ugr.es (J.S.-R.); 4 Laboratorio de Ciencias de la Actividad Física, el Deporte y la Salud, Universidad de Santiago de Chile, USACH, Santiago 7500618, Chile; antonio.garcia.h@usach.cl; 5 Facultad de Ciencias de la Salud, Universidad San Sebastián, Santiago 8420524, Chile

**Keywords:** bioelectrical impedance analysis, diagnosis, nutritional status, youths

## Abstract

This study aimed to determine thresholds for percentage of body fat (BF%) corresponding to the cut-off values for overweight/obesity as recommended by the International Obesity Task Force (IOTF), using two bioelectrical impedance analyzers (BIA), and described the likelihood of increased cardiometabolic risk in our cohort defined by the IOTF and BF% status. Participants included 1165 children and adolescents (54.9% girls) from Bogotá (Colombia). Body mass index (BMI) was calculated from height and weight. BF% of each youth was assessed first using the Tanita BC-418^®^ followed by a Tanita BF-689^®^. The sensitivity and specificity of both devices and their ability to correctly classify children as overweight/obesity (≥2 standard deviation), as defined by IOTF, was investigated using receiver operating characteristic (ROC) by sex and age groups (9–11, 12–14, and 13–17 years old); Area under curve (AUC) values were also reported. For girls, the optimal BF% threshold for classifying into overweight/obesity was found to be between 25.2 and 28.5 (AUC = 0.91–0.97) and 23.9 to 26.6 (AUC = 0.90–0.99) for Tanita BC-418^®^ and Tanita BF-689^®^, respectively. For boys, the optimal threshold was between 16.5 and 21.1 (AUC = 0.93–0.96) and 15.8 to 20.6 (AUC = 0.92–0.94) by Tanita BC-418^®^ and Tanita BF-689^®^, respectively. All AUC values for ROC curves were statistically significant and there were no differences between AUC values measured by both BIA devices. The BF% values associated with the IOTF-recommended BMI cut-off for overweight/obesity may require age- and sex-specific threshold values in Colombian children and adolescents aged 9–17 years and could be used as a surrogate method to identify individuals at risk of excess adiposity.

## 1. Introduction

Assessment of pediatric body composition is of increasing interest for routine monitoring of treatment efficacy, including weight-loss interventions [1]. The most commonly used measure of adiposity is body mass index (BMI); studies also used the z-BMI standard deviation (SD) scores, a measure relative to weight and adjusted for child age and sex [2]. However, z-BMI does not differentiate between fat mass (FM) and fat-free mass (FFM), and is a poor predictor of body fat [3].

In this context, precise measurement of body composition by sophisticated methods, such as computed tomography, dual-energy X-ray absorptiometry (DEXA), magnetic resonance imaging, and air-displacement plethysmography, cannot be applied in routine clinical practice. These methods are costly, time-consuming, and frequently difficult to access. Other disadvantages of these techniques include limited portability, lack of real-time feedback, repeatability, and accessibility. An alternative method is bioelectrical impedance analysis (BIA). BIA is quick, safe, non-invasive, and is considered one of the most reliable [4,5] and accessible methods of screening body fat [6]. In addition, it has been widely used in hospitals and research areas [7]. Correlation coefficients between parameters of body composition assessed by different BIA devices were highly related to those determined by DXA or magnetic resonance imaging (*r* values between 0.92 and 0.96) [4], but several publications have criticized the accuracy of BIA [5].

Foot-to-foot BIA consumer devices are widely used to assess body composition among children and adolescents, and several studies have investigated the accuracy of single-frequency foot-to-foot BIA devices in assessing body fat [8,9]. The Tanita BF-689^®^ (Tanita Corp., Tokyo, Japan) is a BIA used in many field studies in Latin-America [10], Europe, and North America [11], and with its low cost (less than US$150), it is practical for population-based studies. This device is a foot-to-foot BIA device marketed by Tanita as the world’s first body fat scale for kids providing ‘Food and Drug Administration cleared body fat judgment (in children) 5–17 years’ [12]. A recent study [11] showed that, compared to a DEXA device, the BF-689^®^ is affordable and portable, and suggests that the BF-689^®^ is an efficient means of assessing BF% in elementary school-aged children.

On the other hand, the Tanita BC-418^®^ (Tanita Corp., Tokyo, Japan), a multi-frequency device, was introduced to the world market in the last few years and has begun to be used in clinical settings to detect water and FFM in five segments of the body. In 2015, Meredith-Jones et al. [13] tested its ability to predict a four-component model in children and concluded the instrument was strongly correlated and within the cross-validation criterion (*r* = 0.96) when compared with DXA.

In children and adolescents, body composition assessment appears to be even more challenging, as—depending on the growth and biological maturation—there is a large variation in the different body components (water, protein, minerals, etc.), from birth to adulthood [14,15]. This variation can significantly affect the estimate of FM and FFM, especially in two-compartment models [16,17]. In addition, the South American population has been described as having particular growth, development and childhood body composition characteristics resulting from the intermingling of European, Native American, and African ancestors, so it is difficult to make a clear differentiation between environmental and genetic factors [16]. Thus it seems necessary to study body composition as a priority in primary health care [17].

The Tanita BC-418^®^ is not practical for use in the field due to its high cost. Several large studies in the school environment have used the Tanita BF-689^®^, a cheaper device, therefore it is necessary to determine the predictive ability of this new and convenient method in the Latin-American pediatric population. The body fat (BF%) values associated with the International Obesity Task Force (IOTF)-recommended BMI cut-off for overweight and obesity may require age- and sex-specific threshold values in Colombian children and adolescents aged 9–17 years and could be used to identify individuals at risk of surrogate excess adiposity. Thus, this study aimed to determine thresholds for percentage of BF% corresponding to the cut-off values for overweight/obesity as recommended by the IOTF, using two bioelectrical impedance analyzers (BIA), and described the likelihood of increased cardiometabolic risk in our cohort defined by the IOTF and BF% status.

## 2. Methods

### 2.1. Participants and Study Design

This is a secondary analysis of a cross-sectional study, published elsewhere [18,19,20]. Briefly, this study aimed to examine relationships between physical fitness levels in children and adolescents with cardiometabolic risk factors and (un)healthy habits, during the 2014–2015 school year. The sample consisted of children and adolescents (boys *n* = 4000 and girls *n* = 4000) ages 9–17.9 years. Body composition was randomly performed in 12% of the recruited subjects (*n* = 1165). There were no differences in the study key characteristics (i.e., age, sex distribution, and BMI) between the current study sample and the original FUPRECOL Study (*in Spanish*, ASOCIACIÓN DE LA **FU**ERZA **PRE**NSIL CON MANIFESTACIONES DE RIESGO CARDIOVASCULAR TEMPRANAS EN NIÑOS Y ADOLESCENTES **COL**OMBIANOS) [10,18,19,20] sample (*n* = 8000, all *p* > 0.100). The schoolchildren were of low-middle socioeconomic status (SES, 1–3 defined by the Colombian government) and enrolled in public elementary and high schools (grades 5 and 11) in the capital district of Bogota in a municipality in the Cundinamarca Department in the Andean region. A convenience sample of volunteers was included and grouped by sex and age with one-year increments (a total of nine groups). The Review Committee for Research on Human Subjects at the University of Rosario (Code No. CEI-ABN026-000262) approved all of the study procedures. A comprehensive verbal description of the nature and purpose of the study and its experimental risks was given to the participants and their parents/guardians. This information was also sent to parents/guardians by mail. Written informed consent was obtained from parents and subjects before participation in the study. The protocol was in accordance with the latest revision of the Declaration of Helsinki and current Colombian laws governing clinical research on human subjects (Resolution 008430/1993 Ministry of health).

### 2.2. Anthropometric Measurements

Anthropometric variables were measured in accordance with the International Society for the Advancement of Kinanthropometry guidelines [21]. Variables were collected at the same time in the morning, between 7:00 and 10:00 a.m., following an overnight fast. Body weight was measured in the subjects’ underwear and with no shoes, using electronic scales (Tanita BC-544^®^ and Tanita BF-689^®^, Tokyo, Japan) with a low technical error of measurement (TEM = 0.510% and 0.417%, respectively). Height was measured using a mechanical stadiometer platform (Seca^®^ 274, Hamburg, Germany; TEM = 0.01%). Waist circumference (WC) was measured at the midpoint between the last rib and the iliac crest using a tape measure (Ohaus^®^ 8004-MA, Parsippany, NJ, USA; TEM = 0.086). Body mass index (BMI) was calculated as the body weight in kilograms divided by the square of height in meters. Weight status (i.e., underweight, normal weight, or overweight/obese) was defined according to the IOTF age and sex-specific thresholds for BMI [22].

### 2.3. BIA Measurements

All measurements were taken in a single five-minute individual testing session. BF% on each child was assessed first using the Tanita BC-418^®^ followed by a Tanita BF-689^®^. This testing sequence and the use of separate raters for experimental (Tanita BC-689^®^) and reference (Tanita BF-418^®^) standards insured that raters for the experimental method were blind to reference standard results. For each BIA, sex, age, and height were entered directly into the instrument prior to the impedance measure. Testing was scheduled to allow for a 10–12 h fasting window and participants were asked to void their bladder before testing to optimize BF% assessment accuracy. Subjects’ feet were guided onto the BIA foot sensors by the raters to ensure optimal contact and centralized heel placement. All BIA measurements were completed by a trained investigator according to the device manufacturers’ instructions.

### 2.4. Biochemical Assessments

Blood samples were collected between 6:00 and 8:00 a.m. by two experienced pediatric phlebotomists after at least 12 h fasting. Before the extraction, fasting condition by the child and parents was confirmed. Blood samples were obtained from an antecubital vein, and analyses were subsequently completed within one day from collection. The levels of triglycerides (TG), total cholesterol (TC), cholesterol linked to high-density lipoproteins (HDL-C) and glucose were measured using colorimetric enzymatic methods using a Cardiocheck analyzer. The fraction of cholesterol linked to low-density lipoproteins (LDL-C) was calculated using the Friedewald equation ([LDL-C] = [TC] − [HDL-C] − ([TG]/5)), where TG = concentration of triglycerides. The precision performance of these assays was within the manufacturer’s specifications. Blood pressure was measured using an electronic oscillometric device, (Riester Ri-Champion model, Jungingen, Germany) according to the standard American Heart Association protocol for children [23] after being seated in a quiet room for 10 min with their back supported and feet on the ground. Two blood pressure readings were taken with a 10 min interval of quiet rest. Before blood pressure session monitoring, the accuracy of the device was tested against a standard mercury sphygmomanometer in a random sub-sample (*n* = 25) to ensure that there was no consistent difference of >10 mmHg in measured blood pressure; and inter-observer variability was *R* = 0.96.

### 2.5. Cardiometabolic Risk Assessment

At present, adequate guidelines are lacking to delineate the structure and norms for risk factor stratification of cardiometabolic risk in pediatrics [23]. Therefore, we calculated a cardiometabolic risk index (CMRI) as the sum of the age-sex standardized scores of WC, TG, HDL-C, glucose, systolic (SBP), and diastolic blood pressure (DBP). The HDL-C value was then multiplied by −1 as this is inversely related to cardiovascular risk. An age adjusted continuous CMRI (composite z-score) was calculated for each participant as follows:

CMRI = z-WC + z-triglycerides + z-HDL-C + z-glucose + z-SBP + z-DBP

Sex-specific high-risk cardiometabolic phenotypes were defined as ≥75th percentile of the CMRI. The higher the value in the CMRI, the higher the cardiovascular risk [24,25,26].

### 2.6. Sexual Maturation

Sexual maturation was classified based on Tanner staging [27], which uses self-reported puberty status to classify participants into stages I to V [28]. Each volunteer entered an isolated room where they categorized the development of their own genitalia (for boys), breasts (for girls), armpits (for boys), and pubic hair (for both genders) using a set of images exemplifying the various stages of sexual maturation. The reproducibility of our data reached *R* = 0.78. The data were recorded on paper by the FUPRECOL evaluators.

### 2.7. Statistical Analysis

Descriptive statistics were reported as means ± SD. Two sided *t tests* and Pearson χ^2^ tests were used to analyze the differences in means and proportions between groups. A *Pearson* correlation analysis between adiposity measurements and anthropometrics’ indicators was performed. Sensitivity and specificity of the Tanita BC-418^®^ and Tanita BF-689^®^ and ability to correctly classify children as overweight/obesity (≥2SD) defined by IOTF [22] was investigated with Receiver operating characteristic curves (ROC). Cut-off values were derived mathematically from the ROC curves, using the point on the ROC curve with the lowest value for the formula: (1-sensitivity)^2^ + (1-specificity)^2^. The positive likelihood ratio LR (+) and the negative likelihood ratio LR (−) were also determined. Multivariable logistic regression was used to calculate odds ratios and 95% confidence intervals (ORs 95% CIs) for increased CMRI for BF%-BIA (cut-offs) and BMI categories (≥2SD), adjusting for Tanner maturation. All analyses were stratified by sex- and age (9 to 11 years; 12 to 14 years; and 15 to 17 years). The areas under the ROC curves (±SE) were calculated for each BIA device and compared [29]. Analyses were conducted for boys and girls separately in three groups (9 to 11 years; 12 to 14 years 15 to 17 years). All analyses were performed using the Statistical Package for Social Sciences (v. 22.0 for Windows, Chicago, IL, USA), and the level of significance was set to 0.05.

## 3. Results

Descriptive characteristics of participants stratified by sex and age groups are presented in Table 1, showing the demographic descriptive statistics of the sample. The final sample had a mean age (standard deviation (SD); (range)) of 13.0 years (2.3 (9 to 17)) and comprised slightly more females (55%), compared to males (45%). Independently of age, girls had lower levels of BMI, WC, FFM, and lean mass than boys (*p* < 0.05).

Correlation analyses between adiposity measurements and anthropometrics indicators by sex are shown in Table 2. In the three age groups and both sexes the BMI and WC were significantly correlated with both BF%-BIA measures (Tanita BC-418^®^ and Tanita BF-689^®^).

**Figure 1:**
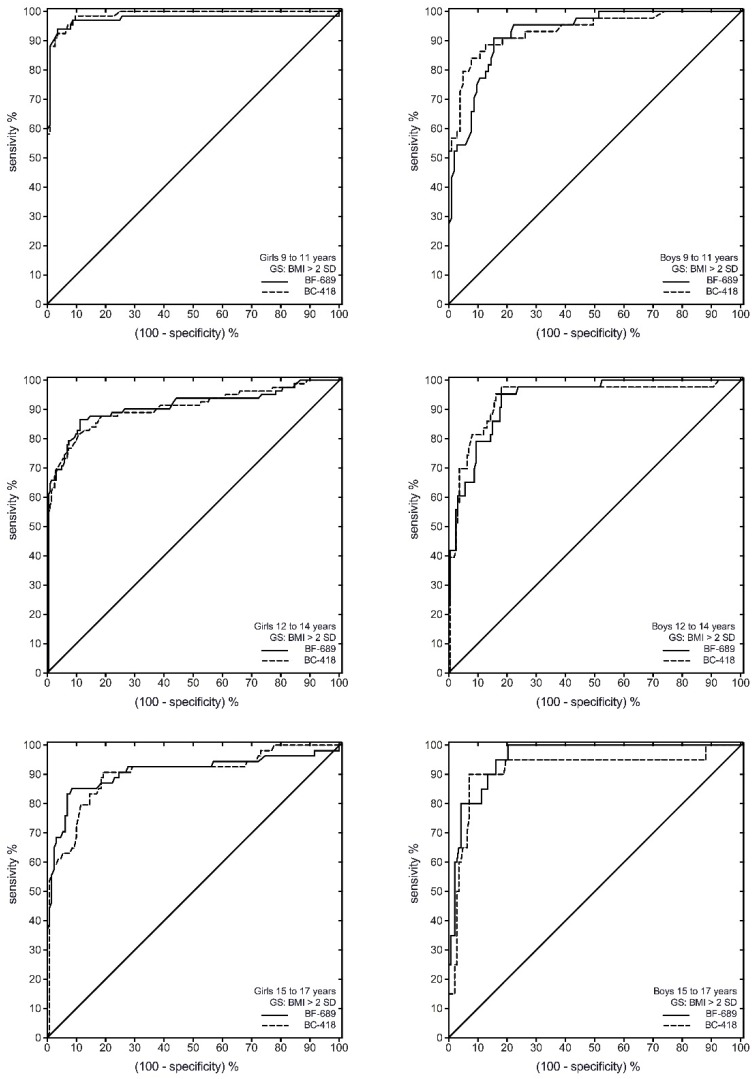
Receiver operating characteristic (ROC) curve of the BF% for ability to correctly classify children as overweight/obesity (≥2SD) defined by IOTF between Tanita BC-418^®^ and Tanita BF-689^®^ among Colombian children and adolescents. GS, gold standard.

ROC analysis showed that both Tanita BC-418^®^ and Tanita BF-689^®^ had a high discriminating power to detect IOTF overweight/obesity (Figure 1 and Table 3). The cut-off for overweight/obesity for boys varied by age, with older boys generally having a lower cut-off, ranging from 15.8 to 20.6 for the Tanita BF-689^®^ and 16.5–21.1 for the Tanita BC-418^®^. The cut-off for overweight/obesity for girls also varied by age, with younger girls generally having a lower cut-off, ranging from 23.9 to 26.6 for the Tanita BF-689^®^ and 25.2–28.5 for the Tanita BC-418^®^ (Table 3).

**Figure 2:**
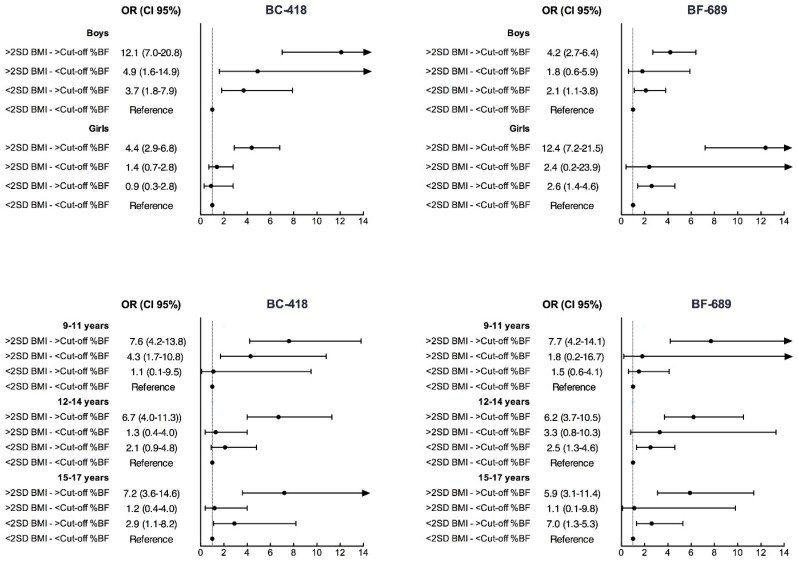
Adjusted odds ratios (OR), with 95% confidence intervals, of being at increased cardiometabolic risk with BF% above and below sex- and age-specific thresholds and BMI (<2SD or ≥2SD).

Both adjusted odds of increased cardiometabolic risk were significantly higher for all groups when compared with <BF% cut-off and BMI < 2SD as the reference group (Figure 2). As expected, the highest odds were found when individuals with BF% cut-off and BMI ≥ 2SD were compared with the reference group. Odds of increased cardiometabolic risk were higher when comparing individuals who were above the ≥ BF% cut-off with BMI ≥ 2SD with the reference group than when comparing those below the BF% threshold and BMI < 2SD with the reference group. Similar relationships were found in both sexes in all age groups. Finally, Table 4 shows differences between the AUC for each BIA device, and no difference was detected in any age group or sex.

## 4. Discussion

This paper provides age- and sex-specific BF% reference thresholds for discriminating overweight/obesity in Colombian children and adolescents using two BIA devices, one of them a low-cost alternative for possible use in large studies and the school environment. These thresholds also allow the opportunity to use BF% along with BMI in epidemiologic research, fitness assessment, and clinical practice.

These results, which are coherent with human sexual dimorphism, agree with the results obtained by Wang et al. [30] for a population of 255 Chinese children and adolescents. Also, the results are in consonance with those of Pan et al. [31] for a sample of soldiers in the graduating class of a Chinese military college. Nevertheless, the comparison of our sample population with samples in China should be interpreted with caution since, as highlighted by Freedman [32], children and adolescents show marked differences in their body composition, particularly in regard to body fat, depending on their race or ethnic group. Therefore, the origin of the samples must be taken into account to establish an appropriate cut-off for defining excess of fat [33] and variations in criteria among various ethnic populations should be considered in evaluating obesity in children and adolescents [34]. Despite the fact that South America has been described as having particular growth, development, and childhood body composition characteristics resulting from the intermingling of European, Native American, and African ancestors, there are no generally accepted BF% cut-offs for excess of adiposity in Latin-American children and adolescents. The lack of definitive standards may be due, in part, to the lack of appropriate reference data to characterize growth and maturation. In the present study, values from the IOTF-recommended BMI cut-off (≥2SD) were used to identify subjects with an excess of adiposity, taking into consideration age and sex. The correlation analysis between adiposity measurements and anthropometric indicators, according to sex and age groups, showed that the BMI and WC were significantly correlated with both BF%-BIA measures (Tanita BF-689^®^ and Tanita BC-418^®^). Therefore, confirming Kabiri et al.’s [11] results, our findings show that the Tanita BF-689^®^ gives an excellent performance in diagnosing overweight and obesity as reflected in the estimate of the BF% in the Latin-American pediatric sample studied.

The AUC values for the overweight and obesity ROC analyses indicate that BF% has high diagnostic capabilities to identify youths with excess adiposity. As shown here and in previous studies, there are distinct age- and sex-associated variations in BF%, presumably due to the natural variations in BF% during development [35]. Taylor et al. [36] reported that BF% values associated with BMI classifications of overweight and obesity vary considerably with age in growing youth, particularly in girls; classifying children and adolescents as overweight ranged from 18% to 23% in boys and from 20% to 34% in girls (3–18 years old). Therefore, our BF% cut-off values tended to be lower than the published BF% cut-off values in Caucasian [36] and Korean youths (cut-off for obesity) [37]. As our sensibility analysis shows, proposed BF% cut-offs for overweight/obesity are related with higher cardiometabolic risk in both sex and age groups. Several international studies have used metabolic syndrome as the criterion to identify corresponding healthy ranges of BF% [37,38,39,40]. A large study of children and adolescents in the US by the National Health and Nutrition Examination Survey reported that BF% thresholds of 22.3% and 35.1% in boys and 31.4% and 38.6% in girls were found to be indicative of low and high metabolic syndrome risk. Another study from The Bogalusa Heart study indicated that skinfold-derived values of 25% BF in boys and 30% BF in girls were predictive of being in the upper quintile for SBP, DBP, total cholesterol, and lipoprotein fractions. Dwyer and Blizzard [39], identified thresholds of 20% BF for boys and 30% BF for girls that were predictive of having higher SBP and lower HDL-C. Our cut-offs in general were lower both in boys and girls, even taking into account an older-age group (15–17 years old), therefore with the FITNESSGRAM cut-offs, the prevalence of metabolic risk in the Latin-American population is often underestimated.

An important issue highlighted by our study was the use of different instruments in BF% measurement. BIA has been used as a field method for body composition measurement due to its non-invasiveness, ease of use, and relatively low cost—currently BIAs are used in school-based health-related fitness testing programs. Both BIA devices provided similar body fat ranges and no difference was detected in any age group or sex according to AUC classification. Other studies of geographically different populations [41] found that the BIA tended to underestimate the BF% in children and adolescents. This evidently questions its validity as the main parameter used to establish cut-off points that define overweight and obesity in a pediatric population. Other research, such as that of Bohn et al. [42], who studied 3327 children and adolescents in Germany, Austria, and Switzerland, also states that the BF% has a lower capacity to predict cardiovascular risk than the BMI in the pediatric population. However, regardless of this consideration, the establishment of BF% cut-off values for age and sex could be of great clinical usefulness since such values would help to estimate real loss of BF. This would be an incentive for pediatric patients and enhance their performance in educational programs to improve and control their weight. However, prospective studies are needed to examine this assumption.

This study had some limitations. First, it includes participants from only a single region in Colombia from public schools in one city; therefore, inferences to all Colombian children and adolescents should be made cautiously. However, Bogotá is the largest urban center in Colombia, comprising about 15% of the country’s population. It includes a mix of locally born residents and populations from other regions of the country that relocate there, with a large racial and cultural diversity. Our subjects were a convenience sample and may not be representative of the populations from which they were recruited. Second, we have not considered the potential impact of recognized determinants such as socio-economic, dietary, and physical-activity patterns, and ethnic factors that modulate growth and levels of adiposity. Another limitation is that this study did not include assessments of students attending private schools. This is because the study was deployed in collaboration with the Bogotá District Education Department, which only has jurisdiction among public schools. However, the public system constitutes the majority of schools provided in the city, with 85% of school-age children enrolled in the city public school system. Therefore, inferences to all Bogotan or Colombian children and adolescents should be made cautiously. This is an area for future research. Nevertheless, such limitations do not compromise the results obtained when validating our results.

This study also has various strong points that should be highlighted. The first is the large size of the population sample. Also, BF% may have a different relation than BMI to weight-related clinical conditions such as metabolic syndrome [43].

## 5. Conclusions

In conclusion, our findings provide BF% thresholds that correspond to the risk of excess of adiposity based on traditional IOTF-BMI cut-off values, one of them using a low-cost alternative device for possible use in large studies and the school environment. Our thresholds can be used as provisional guidelines for recommending ranges of BF% that indicate a minimal risk of developing morbidity associated with excess adiposity in epidemiologic research, fitness assessment, and clinical practice. Moreover, the data suggest that preventive efforts should be focused on those with few ideal health behaviors or factors, and should target early development of cardiovascular disease to reduce the risk of premature health problems.

## Figures and Tables

**Table 1:** Characteristics of school children by sex and age.

	Boys (*n* = 550)			Girls (*n* = 615)		
	9–11 Years (*n* = 159)	12–14 Years (*n* = 233)	15–17 Years (*n* = 158)	9–11 Years (*n* = 175)	12–14 Years (*n* = 273)	15–17 Years (*n* = 167)
Age (years)	9.9 (0.8)	13.2 (0.8)	15.6 (0.7)	10.0 (0.8)	13.2 (0.8)	15.7 (0.8) ^b^
Body mass (kg)	34.8 (8.0)	48.1 (10.1)	58.2 (9.0) ^d^	35.5 (8.3)	47.3 (7.9)	53.6 (8.3) ^b^
Height (cm)	137.7 (8.6)	156.2 (10.0) ^c^	167.5 (7.0) ^d^	138.9 (8.5)	152.6 (6.5)	156.6 (5.9) ^b^
Body mass index (kg/m^2^)	18.2 (2.7)	19.6 (3.1) ^d^	20.7 (2.9) ^d^	18.2 (2.9)	20.3 (2.9)	21.9 (3.2) ^b^
Waist circumference (cm)	62.1 (7.1) ^d^	65.9 (6.8) ^d^	69.6 (7.1) ^d^	60.1 (7.0)	63.1 (6.7)	67.3 (6.7) ^b^
Weight status, *n* (%)						
Underweight	10 (6.8)	34 (16.6)	25 (15.3)	33 (18.3)	43 (15.1)	27 (14.7)
Normal	93 (63.3)	128 (62.4)	118 (72.4)	80 (44.4)	160 (56.1)	103 (56.0)
Overweight	26 (17.7)	30 (14.6)	17 (10.4)	45 (25.0)	68 (23.9)	44 (23.9)
Obesity	18 (12.2)	13 (6.3)	3 (1.8)	22 (12.2)	14 (4.9)	10 (5.4)
BIA Measures						
Fat-free Mass (kg)	27.5 (5.3)	40.1 (7.6) ^d^	49.4 (6.7) ^d^	26.8 (4.7)	34.9 (4.2)	39.5 (4.0) ^b^
Lean Mass (kg)	26.0 (5.1)	37.9 (7.4) ^d^	47.1 (5.8) ^d^	25.4 (4.5)	33.0 (4.2)	37.5 (4.0) ^b^
% Body Fat (Tanita BC-418^®^)	18.5 (6.4) ^d^	15.6 (7.1) ^d^	12.9 (5.7) ^d^	22.3 (6.6)	24.3 (5.5)	24.6 (6.8) ^b^
% Body Fat (Tanita BF-689^®^)	20.6 (6.3) ^d^	16.9 (6.9) ^d^	15.2 (6.1) ^d^	23.6 (6.2)	25.5 (6.4)	25.2 (6.1) ^a^

Two-way ANOVA was applied to compare the differences in means between age (^a^ *p* for trend < 0.05, ^b^ *p* for trend < 0.001). Two-sample *t*-tests was used to determined sex differences and age group (^c^ *p* < 0.05, ^d^ *p* < 0.001).

**Table 2:** Adjusted correlations between percentage of body fat (Tanita BF-689^®^ and Tanita BC-418^®^), body mass index, and waist circumference by sex and age-groups.

	9–11 Years	12–14 Years	15–17 Years
*Boys (Tanita BC-418^®^)*			
Body mass index (kg/m^2^)	0.852	0.689	0.739
Waist Circumference (cm)	0.758	0.631	0.521
*Girls (Tanita BC-418^®^)*			
Body mass index (kg/m^2^)	0.928	0.842	0.726
Waist Circumference (cm)	0.841	0.704	0.699
*Boys (Tanita BF-689^®^)*			
Body mass index (kg/m^2^)	0.814	0.752	0.763
Waist Circumference (cm)	0.711	0.684	0.542
*Girls (Tanita BF-689^®^)*			
Body mass index (kg/m^2^)	0.915	0.644	0.696
Waist Circumference (cm)	0.815	0.468	0.765

All values were *p* < 0.001.

**Table 3:** Area under the receiver-operating characteristic curves for ability to correctly classify youths as overweight/obesity (≥2SD) defined by IOTF among Colombian children and adolescents.

	9–11 Years				12–14 Years				15–17 Years			
	Tanita BC-418^®^		Tanita BF-689^®^		Tanita BC-418^®^		Tanita BF-689^®^		Tanita BC-418^®^		Tanita BF-689^®^	
	Boys	Girls	Boys	Girls	Boys	Girls	Boys	Girls	Boys	Girls	Boys	Girls
AUC (95% CI)	0.93	0.97	0.94	0.99	0.93	0.91	0.94	0.91	0.96	0.91	0.92	0.90
	(0.88–0.97)	(0.94–0.00)	(0.89–0.98)	(0.98–0.00)	(0.90–0.97)	(0.86–0.96)	(0.89–0.98)	(0.86–0.95)	(0.92–0.99)	(0.85–0.97)	(0.83–0.99)	(0.84–0.95)
Optimal cut-off	21.1	25.2	20.6	23.9	18.4	27.1	16.8	26.6	16.5	28.5	16.8	27.5
J-Youden	0.75	0.91	0.76	0.89	0.77	0.75	0.80	0.71	0.80	0.77	0.83	0.72
Sensitivity (%)	90.9	94.0	84.1	92.5	95.3	86.6	97.7	81.7	99.8	85.2	90.0	90.7
Specificity (%)	84.5	96.5	92.2	96.5	81.9	88.7	81.9	89.2	79.7	91.5	93.0	80.8
LR (+)	5.86	6.86	0.78	6.43	5.27	7.66	5.40	7.56	4.93	10.02	12.86	4.72
LR (−)	0.11	0.06	0.17	0.08	0.06	0.15	0.03	0.21	0.01	0.16	0.11	0.12

AUC: area under curve; LR (+): positive likelihood ratio; LR (−): negative likelihood ratio.

**Table 4:** Differences between areas under the receiver-operating for each BIA devices.

	Tanita BF-689^®^		Tanita BC-418^®^		Correlation	*z*-Value
	AUC	SE	AUC	SE		
Girls 9–11 y	0.97	0.02	0.99	0.01	0.82	0.447
Girls 12–14 y	0.91	0.02	0.91	0.02	0.86	0.936
Girls 15–17 y	0.91	0.03	0.90	0.03	0.86	0.928
Boys 9–11 y	0.93	0.02	0.94	0.02	0.85	0.779
Boys 12–14 y	0.93	0.02	0.94	0.02	0.86	0.976
Boys 15–17 y	0.96	0.02	0.92	0.04	0.84	0.390

AUC: area under curve; SE: standard error; y: years.
