# Correction: Ramírez-Vélez, R.; et al. Performance of Two Bioelectrical Impedance Analyses in the Diagnosis of Overweight and Obesity in Children and Adolescents: The FUPRECOL Study. *Nutrients* 2016, 8, 575

**DOI:** 10.3390/nu8110705

**Published:** 2016-11-05

**Authors:** Robinson Ramírez-Vélez, Jorge Enrique Correa-Bautista, Javier Martínez-Torres, Katherine González-Ruíz, Emilio González-Jiménez, Jacqueline Schmidt-Rio Valle, Antonio Garcia-Hermoso

**Affiliations:** 1 Centro de Estudios para la Medición de la Actividad Física «CEMA», Escuela de Medicina y Ciencias de la Salud, Universidad del Rosario, Bogotá D.C. 111221, Colombia; jorge.correa@urosario.edu.co (J.E.C.-B.); javiermartinezt@usantotomas.edu.co (J.M.-T.); 2 Grupo de Ejercicio Físico y Deportes, Vicerrectoría de Investigaciones, Universidad Manuela Beltrán, Bogotá, D.C. 110231, Colombia; Katherine.gonzalez@docentes.umb.edu.co; 3 Grupo CTS-436, Adscrito al Centro de Investigación Mente, Cerebro y Comportamiento (CIMCYC), Departamento de Enfermería, Universidad de Granada, Granada 18071, España; emigoji@ugr.es (E.G.-J.); jschmidt@ugr.es (J.S.-R.V.); 4 Laboratorio de Ciencias de la Actividad Física, el Deporte y la Salud, Universidad de Santiago de Chile, USACH, Santiago 7500618, Chile; antonio.garcia.h@usach.cl or antoniogh@unex.es; 5 Facultad de Ciencias de la Salud, Universidad San Sebastián, Santiago 8420524, Chile

We would like to make the following correction to our recently published paper [1]. First, the FUPRECOL study was conducted from 2014 to 2015 in a convenience sample of volunteers and grouped by sex and age with one-year increments (a total of nine groups). In total, 8000 school children from 27 official schools aged 9.0–17.0 years, with valid data for gender and body mass index (BMI) were included in a primary study [2]. In this paper, we analyzed a secondary cross-sectional study through data from the bioelectrical impedance (BIA) analysis. This sample size was randomly performed in one-sixth of the recruited children and adolescents (*n* = 1164, in six official schools). Second, the sample size was 1165 (52.7% girls) which represents 14.5% of the primary sample size study. Third, the match between the participant’s distribution by age groups and gender in the weight status has been corrected (Table 1).

These changes have no material impact on the conclusions of our paper. The manuscript will be updated and the original will remain online on the article webpage. We apologize for any inconvenience caused to our readers.

## Figures and Tables

**Table 1:** Characteristics of school children by sex and age.

Weight Status	Boys			Girls		
	9–11 Years	12–14 Years	15–17 Years	9–11 Years	12–14 Years	15–17 Years
	(*n* = 159)	(*n* = 233)	(*n* = 158)	(*n* = 175)	(*n* = 273)	(*n* = 167)
Underweight	**11** (6.8)	**39** (16.6)	**24** (15.3)	**32** (18.3)	**41** (15.1)	**25** (14.7)
Normal	**101** (63.3)	**145** (62.4)	**114** (72.4)	**78** (44.4)	**153** (56.1)	**94** (56.0)
Overweight	**28** (17.7)	**34** (14.6)	**16** (10.4)	**44** (25.0)	**65** (23.9)	**40** (23.9)
Obesity	**19** (12.2)	**15** (6.3)	**3** (1.8)	**21** (12.2)	**13** (4.9)	**9** (5.4)
